# Acceptability and Feasibility of Sexually Transmitted Infection Testing and Treatment among Pregnant Women in Gaborone, Botswana, 2015

**DOI:** 10.1155/2016/1251238

**Published:** 2016-03-28

**Authors:** Adriane Wynn, Doreen Ramogola-Masire, Ponatshego Gaolebale, Neo Moshashane, Ogechukwu Agatha Offorjebe, Kaitlin Arena, Jeffrey D. Klausner, Chelsea Morroni

**Affiliations:** ^1^Department of Health Policy and Management, Fielding School of Public Health, University of California, Los Angeles, 650 Charles Young Dr. S., 31-269 CHS, P.O. Box 951772, Los Angeles, CA 90095-1772, USA; ^2^Botswana-UPenn Partnership, 244G UB Main Campus, Gaborone, Botswana; ^3^University of Botswana, 4775 Notwane Road, Gaborone, Botswana; ^4^Princess Marina Hospital, Phologolo Extension 9, Gaborone, Botswana; ^5^Charles R. Drew University of Medicine & Science, 1731 E. 120th Street, Los Angeles, CA 90059, USA; ^6^David Geffen School of Medicine, University of California, Los Angeles, 10833 Le Conte Avenue, Los Angeles, CA 90095, USA; ^7^Division of Infectious Diseases, Department of Epidemiology, David Geffen School of Medicine and Fielding School of Public Health, University of California, Los Angeles, 10833 Le Conte Avenue, CHS 13-154a, Los Angeles, CA 90095, USA; ^8^EGA Institute for Women's Health and Institute for Global Health, University College London, 74 Huntley Street, London WC1E 6AU, UK; ^9^Wits Reproductive Health and HIV Institute, University of Witwatersrand, 58 Klein Street, Johannesburg 2001, South Africa

## Abstract

*Introduction*.* Chlamydia trachomatis* (CT),* Neisseria gonorrhoeae* (NG), and* Trichomonas vaginalis* (TV) are curable sexually transmitted infections (STIs) that can cause adverse maternal and birth outcomes. Most countries do not conduct routine testing during antenatal care. We present data on the acceptability and feasibility of testing and treating pregnant women for STIs in an antenatal clinic in Gaborone, Botswana.* Materials and Methods*. We offered CT, NG, and TV testing using self-collected vaginal swabs to eligible pregnant women. Participants received same-day test results. Those who tested positive were given treatment.* Results*. Among the 225 women who were eligible and recruited, 200 (89%) agreed to participate. The median age of our study sample was 30 years; most were unmarried (77%), with a median gestational age of 27 weeks and a 23% HIV prevalence. All participants received their results with at least 72% (*n* = 143) on the same day. Thirty participants (15%) tested positive for an STI, all were treated, and 24 (80%) were treated on the same day.* Conclusion*. The acceptability of STI testing was high, and the intervention was feasible. This study provides support for continued research into STI prevalence, cost-effectiveness, and the association of STIs with adverse maternal and infant outcomes.

## 1. Introduction

The global prevalence of curable sexually transmitted infections (STIs), including* Chlamydia trachomatis*,* Neisseria gonorrhoeae*,and* Trichomonas vaginalis*, remains high, and a more robust commitment to prevention, identification, and treatment is needed [1]. Untreated STIs have been associated with adverse maternal and birth outcomes [1, 2]. Recent research has identified* Chlamydia trachomatis*,* Neisseria gonorrhoeae*,and* Trichomonas vaginalis* as possible causes of preterm labor and birth [3–7]. Further, mother-to-child transmission of HIV may also be associated with other concurrent STIs [8]. One study of HIV-infected women in Tanzania found that coinfection with* Neisseria gonorrhoeae* was associated with a 5.5-fold increased risk of intrauterine HIV transmission [9].

Despite the adverse impact of STIs on pregnancy and birth outcomes, improvements in STI detection with molecular testing, and availability of one-dose oral treatment regimens, few countries have guidelines that recommend routine STI testing and treatment for pregnant women [10–16].

In many low- and middle-income countries, including Botswana, diagnosis of STIs is primarily through a “syndromic approach.” That approach utilizes an algorithm to classify symptoms and clinical signs into STI syndromes and patients are treated with standardized drug regimens [17, 18]. However,* Chlamydia trachomatis*,* Neisseria gonorrhoeae*, and* Trichomonas vaginalis* are often asymptomatic. Previous research has estimated that only 5–30% of women with chlamydial, gonococcal, or trichomonal infections develop symptoms [19–22]. Thus, the syndromic approach lacks sensitivity, missing a large proportion of infections, and lacks specificity, causing pregnant women to be potentially unnecessarily exposed to antibiotics [23, 24]. Finally, as a result of the predominance of the syndromic approach, little is known about the global prevalence of* Chlamydia trachomatis*,* Neisseria gonorrhoeae*, and* Trichomonas vaginalis* infections among pregnant women.

From July to October 2015, we offered testing and treatment for* Chlamydia trachomatis*,* Neisseria gonorrhoeae*, and* Trichomonas vaginalis* in the antenatal care clinic at Princess Marina Hospital in Gaborone, Botswana. Here, we present data on the acceptability and feasibility of testing and treatment among pregnant women.

## 2. Materials and Methods

We conducted a prospective cohort study to determine STI prevalence, treatment uptake, and cure rates among pregnant women receiving outpatient antenatal care at Princess Marina Hospital in Gaborone, Botswana. We offered STI diagnosis using a GeneXpert® system (Cepheid, Sunnyvale, CA) to eligible women receiving care at the clinic during the intervention period. Women were eligible if they were 18 years or older, had a gestational age of less than 35 weeks, were mentally competent to understand the informed consent, and were willing to return to the clinic for follow-up care. The gestational age cut-off and follow-up criteria were selected to help ensure that women testing positive for an STI could return for a test of cure after four weeks and prior to delivery.

Princess Marina, located in Botswana's capital city, is the main government referral hospital for southern Botswana; approximately 85% of all births in Gaborone occur there [25]. After several weeks of shadowing the providers and integrating the study staff into the vitals room of the clinic, the intervention period began on July 9, 2015, and continued through March 2016.

### 2.1. Description of Intervention

**Figure 1:**
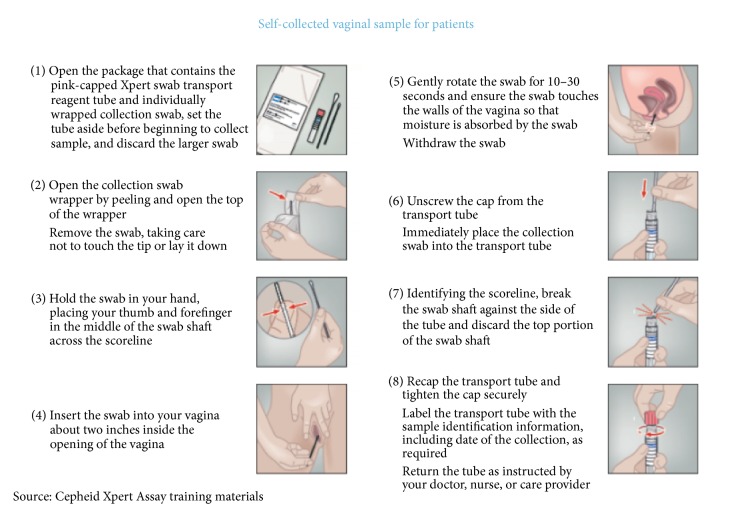


At the start of each clinic day, during the general morning announcements, clinic nurses discussed the availability of STI testing for women meeting the eligibility criteria described above. The announcement included information about the importance of STI diagnosis and treatment during pregnancy. After vitals were collected and recorded by study staff, women were preliminarily screened for eligibility via review of their obstetric records. If eligible, they were provided with additional information about* Chlamydia trachomatis, Neisseria gonorrhoeae, *and* Trichomonas vaginalis* during pregnancy. Women were informed about the global frequency of those infections, their asymptomatic nature, the possible consequences to pregnant women and infants, and the recommended treatments. Women were also informed about the requirements of participation (e.g., they will be asked to provide basic demographic, behavioral, and health history information), the testing procedure via a self-collected vaginal sample, the expected 90-minute wait time, and the need for retest to evaluate the cure rate after four weeks if the results were positive. The procedure for collecting a vaginal sample was explained verbally with the help of Figure 1. Eligible women were also told that if they test positive, they would be advised to inform their partners.

Those consenting were enrolled, instructed on how to self-collect a vaginal swab specimen, and asked to provide demographic, behavioral, and health history information. We collected information on the patients' age, marital status, education level, current gestational age by last normal menstrual period (LNMP) and by ultrasound scan, birth history, HIV status, and prior syphilis diagnosis from the patient obstetric record. We also collected information about prior STI diagnoses and current symptoms that could be associated with an STI via interviews conducted by study staff in Setswana.

The specimens were tested by trained study staff for* Chlamydia trachomatis*,* Neisseria gonorrhoeae*, and* Trichomonas vaginalis* infections using a GeneXpert system (Cepheid, Sunnyvale, CA). Xpert provides 90-minute detection and differentiation of* Chlamydia trachomatis* and* Neisseria gonorrhoeae* and 59 minutes for* Trichomonas vaginalis*. Performance evaluations have found that the sensitivity for the Xpert* Chlamydia trachomatis* test in females using a vaginal sample is 98.7% (95% CI: 93.1–100),* Neisseria gonorrhoeae* is 100.0% (95% CI: 87.3–100), and* Trichomonas vaginalis* is 100% (95% CI: 75.3–100.0) [26–28]. The specificity for the Xpert* Chlamydia trachomatis* test in females using a vaginal sample is 99.4% (95% CI: 98.9–99.7),* Neisseria gonorrhoeae* is 99.9% (95% CI: 99.6–100), and* Trichomonas vaginalis* is 100% (95% CI: 78.1–100.0) [26–28]. All specimens were tested on-site in the antenatal clinic vitals room.

Women were contacted about their results on the same day as testing, either in person or by telephone. If they tested positive, same-day treatment was provided before the women left the clinic. Women who tested positive were given stat doses of directly observed therapy: 1 g of oral azithromycin for chlamydial infection; a 250 mg intramuscular injection of ceftriaxone in the buttocks and 1 g oral azithromycin for gonococcal infection (one person was not treated with azithromycin); and 2 g of oral metronidazole for trichomonas infection.

Those who tested positive were advised to inform their partner(s) and given the option of bringing their partner(s) with them to the antenatal clinic for treatment or giving their partner(s) a contact sheet with instructions to receive treatment at a clinic of their choosing. Additionally, for those who tested positive, a test of cure to ensure clearance of the infection was provided during their standard clinic follow-up appointment, which typically occurs after four weeks. Testing and treatment were free of charge.

The institutional review boards at the University of Botswana, the Botswana Ministry of Health, Health Research Development Committee, and Princess Marina Hospital approved the study protocol. The University of California, Los Angeles, approved analyses using deidentified data.

## 3. Results and Discussion

### 3.1. Acceptability of Intervention

Over the course of 12 weeks of intervention availability, we assessed 728 pregnant women for eligibility. Two hundred and seventy-three (38%) met eligibility criteria and were offered the option of participation. The main reasons for ineligibility were having a gestational age >34 weeks (*n* = 371, 82%) and not planning to receive additional care at Princess Marina (*n* = 62, 14%).

Among the 273 eligible women, 83% (*n* = 225) were offered participation in the intervention. We were unable to approach 100% of the eligible women largely due to study staff capacity. Occasionally, the clinic experienced a lack of running water or electricity, which temporarily caused enrollment to halt.

**Figure 2:**
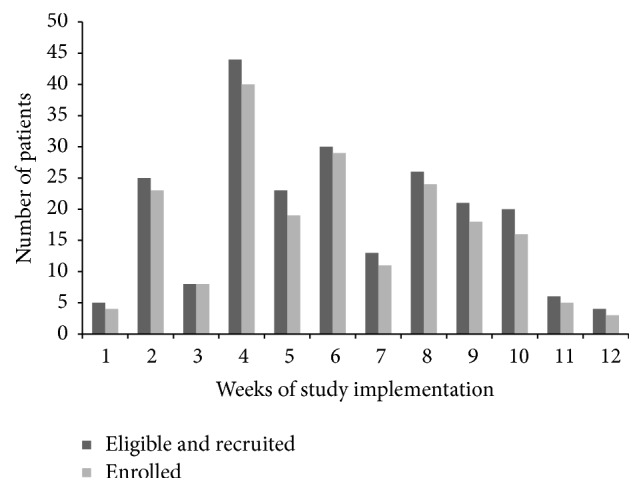
Number of patients eligible, recruited, and enrolled by week in the* Chlamydia trachomatis*,* Neisseria gonorrhoeae*, and* Trichomonas vaginalis* study in Gaborone, Botswana, 2015.

Among the 225 women who were eligible and offered participation, 200 (89%) accepted. Reasons for nonacceptance of the intervention were lack of time (*n* = 10, 40%) and not wanting additional testing (*n* = 2, 8%). Two (8%) gave no reason and four (16%) said that they would return later to test and did not.  Figure 2 illustrates eligibility and acceptability over the course of the first 12 weeks of enrollment. Week 1 included two pilot days and weeks 3, 11, and 12 had low numbers of participants because they contained fewer than four days of implementation each week. The clinic volume was highest on Mondays and Tuesdays and lowest on Thursdays and Fridays. Additionally, during week 9 the clinic experienced water shortages and we were unable to enroll new patients.

Table 1 shows the characteristics of women who participated in the intervention. There were no differences in the demographic and reproductive health data collected for the women who chose not to participate (data not shown).

### 3.2. Feasibility

Among all women who were eligible and consented to participate in the intervention, 100% were successfully tested for* Chlamydia trachomatis*,* Neisseria gonorrhoeae*, and* Trichomonas vaginalis* and given their results. One sample was retested because the temperature of the Xpert machine was above the threshold. Most (*n* = 143, 72%) were given their results in person before leaving the clinic on the day of testing. However, some (*n* = 57, 29%) were contacted by telephone, on the same day, after leaving the clinic because they had completed their clinic visit (the average wait time in clinic is 45 minutes) and could not wait for their STI results (90-minute total testing time).

The prevalence of* Chlamydia trachomatis*,* Neisseria gonorrhoeae*, or* Trichomonas vaginalis* in this sample was 15% (*n* = 30), with* Chlamydia trachomatis* at 10% (*n* = 20),* Trichomonas vaginalis* at 5% (*n* = 10), and* Neisseria gonorrhoeae* at 1.5% (*n* = 3). Among the 30 women who tested positive for an STI, six (20%) received their results over the phone and needed to return to the clinic for treatment. All women who tested positive for an STI (100%) were successfully treated, most immediately (80%).

## 4. Discussion

We implemented an STI testing intervention for pregnant women in the antenatal clinic of Princess Marina Hospital in Gaborone, Botswana. We found that acceptability of the intervention was high, and, as measured by the large proportion of patients who received their results on the same day as testing and the number of positives that were treated, the intervention was also feasible. All enrolled women were able to provide an adequate, self-collected vaginal sample and receive results either in person or by phone.

The results from this study are comparable to other acceptability and feasibility studies, including a recent intervention in Peru where uptake of self-collected vaginal swabs for* Chlamydia trachomatis* testing among pregnant women was 94% (600/640) [29]. Further, one study from two family planning clinics in France found the rate of Xpert assay success to be 98.3% on the first attempt using endocervical swab specimens, and the mean time of results was 2.5 hours (range: 1–5 hours) [30].

There are limitations to our study. Women were recruited from a single site, Princess Marina Hospital antenatal clinic, which provides routine antenatal care and also serves as a referral clinic for women with high-risk pregnancies. While nearly a quarter of our sample reported that their appointment was a routine check-up, the remainder were referred for high-risk conditions. The most common referral condition was high blood pressure. Thus, the results are not necessarily generalizable to other antenatal settings, including local clinics. Further, because the Princess Marina antenatal clinic is a referral center, most women had a gestational age greater than 34 weeks and were ineligible for our study. In order to increase efficiency, future studies should consider expanding enrollment to antenatal clinics attended by women earlier in their pregnancies.

Additionally, the small sample size included in this study is not powered to detect precise estimates of* Chlamydia trachomatis*,* Neisseria gonorrhoeae*, or* Trichomonas vaginalis*. However, it is encouraging to note that the prevalences of* Chlamydia trachomatis* and* Neisseria gonorrhoeae* in our sample are similar to those identified by Romoren et al., who found in a study of 703 antenatal care attendees in Gaborone, Botswana, between October 2000 and February 2001, that 8% and 3% were positive for* Chlamydia trachomatis* and* Neisseria gonorrhoeae*, respectively [31, 32]. Further, the HIV prevalence of 23% in our sample is similar to a 2013 national survey of antenatal care attendees, which found a 20.5% prevalence of HIV among 143,037 women tested during an antenatal care visit [33]. Although our population is not representative of all pregnant women in Botswana, the results of this intervention provide evidence of uptake and feasibility using a busy, high-volume antenatal clinic in Gaborone.

Further, the intervention was run by a team of trained, dedicated study staff and was not fully integrated into the clinic using clinic staff. The study team assisted with vitals collection, providing informed consent, explaining the sample collection procedure, interviewing patients in Setswana, performing sample processing, running the GeneXpert system, and calling over one-quarter of the participants to give them their results. Calling patients with results and ensuring that some return for treatment were time-consuming. In order to facilitate same-day results and treatment, it may be necessary to decrease the already rapid testing period of 90 minutes. Further, it is clear that busy clinics would require staff dedicated to STI testing in order to ensure sustainability.

Despite those limitations, it is important to note that our intervention was able to identify and treat infections that likely would have been missed using the syndromic approach. For example, Romoren et al. estimated that the syndromic approach's sensitivity is only 49% for identifying* Chlamydia trachomatis* and the probability that these diagnosed women are prescribed treatment for* Chlamydia trachomatis* in Botswana is 85% [23, 34]. Thus, testing and treating STIs in pregnant women will identify more cases; however, more evidence is needed to demonstrate that routine STI testing during pregnancy is cost-effective.

## 5. Conclusion

We implemented an STI test and treat intervention for* Chlamydia trachomatis*,* Neisseria gonorrhoeae*, and* Trichomonas vaginalis* in the antenatal care clinic at Princess Marina Hospital in Gaborone. The acceptability of the STI testing was high, and the intervention also proved feasible. This study provides support for continued research into STI prevalence and correlates, partner notification and treatment, cost-effectiveness, and the association of STIs with adverse maternal and infant outcomes. Finally, the results may inform policy, guidelines, and decisions to make STI testing routinely available to pregnant women in Botswana and other low- and middle-income countries.

## Figures and Tables

**Table 1:** Characteristics of study participants enrolled in the *Chlamydia trachomatis, Neisseria gonorrhoeae,* and *Trichomonas vaginalis* study in Gaborone, Botswana, 2015 (*n* = 200).

	*n*	%
Total	200	100
Age, median (IQR)	30	(26–35)
18–24 years	36	18
25–29 years	58	29
30–34 years	58	29
>35 years	48	24
Marital status		
Single	152	76
Married	46	23
Widowed	0	0
Divorced	0	0
Missing	2	1
Education		
None/primary	5	3
Junior secondary	52	26
Senior secondary	65	33
Tertiary	74	37
Missing	4	2
Gestational age weeks (LNMP), median (range)	27	(5–35)
HIV status		
Positive	45	23
Negative	153	77
Unknown	2	1
Prior pregnancies, median (range)	2	(1–11)
Previous births, median (range)	1	(0–7)
Prior STI diagnosis during this pregnancy		
No	163	82
Yes	27	14
Unknown	6	3
Missing	4	2
Positive for an STI	30	15
Positive for *Chlamydia trachomatis*	20	10
Positive for *Neisseria gonorrhoeae*	3	1.5
Positive for *Trichomonas vaginalis*	10	5
Dual STIs	3	1.5
Triple STIs	0	0

Notes: LNMP stands for last normal menstrual period; IQR stands for interquartile range. Percentages may not add up to 100 due to rounding.
